# Pesticide thiram exposure alters the gut microbial diversity of chickens

**DOI:** 10.3389/fmicb.2022.966224

**Published:** 2022-09-08

**Authors:** Zhiwen Wu, Rongsheng Su

**Affiliations:** College of Veterinary Medicine, South China Agricultural University, Guangzhou, China

**Keywords:** thiram, gut microbiota, liver metabolism, chicken, pesticide

## Abstract

Thiram is a major dithiocarbamate pesticide commonly found in polluted field crops, feed, and rivers. Environmental thiram exposure has been demonstrated to cause angiogenesis and osteogenesis disorders in chickens, but information regarding thiram influences on gut microbiota, apoptosis, and autophagy in chickens has been insufficient. Here, we explored the effect of thiram exposure on gut microbiota, apoptosis, and autophagy of chickens. Results demonstrated that thiram exposure impaired the morphology and structure of intestinal and liver tissues. Moreover, thiram exposure also triggered liver apoptosis and autophagy. The gut microbiota in chickens exposed to thiram exhibited a significant decline in alpha diversity, accompanied by significant shifts in taxonomic compositions. Bacterial taxonomic analysis indicated that thiram exposure causes a significant reduction in the levels of eight genera, as well as a significant increase in the levels of two phyla and 10 genera. Among decreased bacterial genera, seven genera even cannot be observed in the thiram-induced chickens. In summary, this study demonstrated that thiram exposure not only dramatically altered the gut microbial diversity and composition but also induced liver apoptosis and autophagy in chickens. Importantly, this study also conveyed a key message that the dysbiosis of gut microbiota may be one of the major pathways for thiram to exert its toxic effects.

## Introduction

The intestine is the main organ for nutrient digestion and absorption and colonizes trillions of microorganisms (Yang et al., 2018). According to statistics, the human intestine inhabits over 10^14^ microorganisms, about 10 times the total amount of human cells (Koboziev et al., 2014). Mounting evidence indicated that gut microbiota played essential roles in immunity, intestinal homeostasis, and epithelium differentiation (Liu et al., 2020; Xiang et al., 2020). Moreover, numerous investigations also revealed the positive regulation roles of gut microbiota in intestinal barrier function, metabolism, and host health (Cani and Delzenne, 2009; Dong et al., 2020). Some bacteria have the ability to restrict the proliferation of pathogenic and opportunistic pathogens in the intestine by producing beneficial metabolites, which was considered a vital barrier against pathogen infection (Wang et al., 2018a). Although intestinal microorganisms reside in the intestine, they may cause systemic effects. Numerous studies provided supporting evidence that gut microbiota was a central or driving factor of many diseases, injuring both near and far organ systems (Acharya and Bajaj, 2021). Gut microbial alterations may extend their detrimental influences beyond the intestine and impair liver and brain (Albhaisi et al., 2020). However, gut microbial homeostasis is easily affected by many factors, such as stress, antibiotics, heavy metal, and pesticide (Kakade et al., 2020). Early studies revealed that gut microbial alternations were associated with many diseases, including diarrhea, diabetes, obesity, and even colorectal cancer (Frazier et al., 2011; Wang et al., 2018b).

Thiram (tetramethyl thiuram disulfide), a broad-spectrum antibacterial pesticide, is widely used in field crops, such as wheat, corn, cotton, and multiple vegetables (Zhang et al., 2018). However, thiram has been demonstrated to be toxic to many animals, including chickens, goats, mice, and fish (Oruc, 2010; Huang et al., 2018). Previous research has indicated that thiram significantly could stimulate the respiratory tract, skin, and gastrointestinal mucosa and inhibit the formation of white blood cells (Walia et al., 2009). Moreover, long-term thiram exposure also caused dysfunction of the central nervous system, internal organs, and endocrine system (Oruc, 2010). Recent studies on thiram toxicity demonstrated that thiram exposure could decrease the quality of the chicken and induce lipid metabolism disorder (Kong et al., 2020). Additionally, other studies indicated that exposure to thiram significantly altered the biochemical indices of liver function and induced bone disease by inhibiting the development of chondrocytes (Mehmood et al., 2019). However, the relationship between thiram exposure and gut microbiota of chickens remains scarce. Herein, we dissected the shifts of gut microbiota in thiram-induced chickens.

## Materials and methods

### Animal experiments

A total of 40 one-day-old healthy Arbor Acres (AA) chickens (with similar original weight) were acquired for animal experiments. The purchased chickens were shifted to the animal room and maintained at a recommended illumination (23 h/1 h light/dark cycle), humidity: (53–57%), and ambient temperature (33–35 °C). The feeding was managed as per the standards previously described (Zhang et al., 2019). All chickens were randomly divided into control (CI) and thiram-treated (TI) groups, with 20 chickens per group. Moreover, chickens in the thiram-exposed group were provided with the same diet as controls but with the addition of thiram (50 mg/kg) in feed as described by previous studies from days 3 to 7 (Yao et al., 2018; Mehmood et al., 2019; Waqas et al., 2019; Zhang et al., 2019). All the chickens were euthanized on day 18 of the experimental study. Afterward, the different intestinal segments were knotted utilizing a cotton rope and separated from mesentery. Meanwhile, the livers were removed from all chickens.

### Histological examination

The collected intestinal and liver tissues were fixed immediately in 4% paraformaldehyde for subsequently preparing histological sections. The specific methods and details of hematoxylin and eosin (H&E) stains were conducted according to previous research (Mehmood et al., 2019).

### RNA extraction and RT-qPCR

Total RNA of liver was extracted using TRIzol reagent as per the efficient RNA extraction method. Subsequently, the isolated RNA was reverse-transcribed into cDNA based on the manufacturer's guidelines. The RT-qPCR was conducted in Step One-PlusTM Real-Time PCR System (Applied Biosystems). The reaction condition and mixture specification were determined as described previously (Mehmood et al., 2019). The relative expression of each gene was calculated with 2^−ΔΔ^CT method and normalized to GAPDH expression. Related primers used in this experiment, such as Atg5, Bak1, Bax, Bcl2, Beclin1, Casp3, Lc3b, and P53, are shown in Supplementary Table S1.

### Western blot analysis

The collected liver was homogenized and centrifuged at 12,000 rpm for 10 min to obtain the supernatant for evaluating total protein concentration. The total protein concentration was assessed by the Coomassie Brilliant Blue g-250 method. Western blot analysis was performed based on the method of previous studies. The main antibodies used in this study were Atg5, Bax, Cytc, Beclin1, Lc3b, and P62. Data were indicated as the protein normalized to GAPDH expression.

### DNA extraction and illumine MiSeq sequencing

All frozen intestinal samples were thawed on ice and homogenized, and the homogenized samples (approximately 200 mg) of each intestinal segment were applied to total genomic DNA extraction based on the manufacturer's protocol. Subsequently, the quality evaluation (integrality, purity, and concentration) of extracted DNA was performed. The specific primers (338F: ACTCCTACGGGAGGCAGCA and 806R: GGACTACHVGGGTWTCTAAT) with adaptors synthesized as per the 16S rRNA conserved regions were used to amplify the V3/V4 hypervariable regions. PCR amplification procedure was performed in triplicates, and reaction conditions and volume were based on previous studies. Afterward, the obtained amplified products were subjected to quality evaluation, recycle target fragment, fluorescent quantitation, and purification. The final libraries were subjected to sequencing following the standard protocols.

### Bioinformatics and statistical analysis

The obtained raw reads were subjected to filter, identify, and remove primer sequences by Trimmomatic (v0.33) and Cutadapt software (1.9.1) to obtain clean reads that do not contain primer sequences. The clean reads of each intestinal sample were performed double-ended sequence splicing using Usearch software (v10), and then the spliced data were filtered by length based on the length range of different areas. The final effective reads were obtained after identifying and removing the chimera sequence using UCHIME software (v4.2). The obtained effective reads were clustered as operational taxonomic units (OTUs) as per 97% similarity. Multiple diversity indices were generated according to the abundance distribution of OTUs in different samples to evaluate the gut microbial diversity. PCoA was employed to visualize the gut microbial difference between control and thiram-exposure groups. Differentially represented microbial taxa between both groups were analyzed utilizing the LEfSe and Metastats analysis. GraphPad Prism (v8.0) was used for performing statistical analysis. Probability values (means ± SD) <0.05 were considered statistically significant.

## Results

### Histopathological observation of intestine and liver

**Figure 1:**
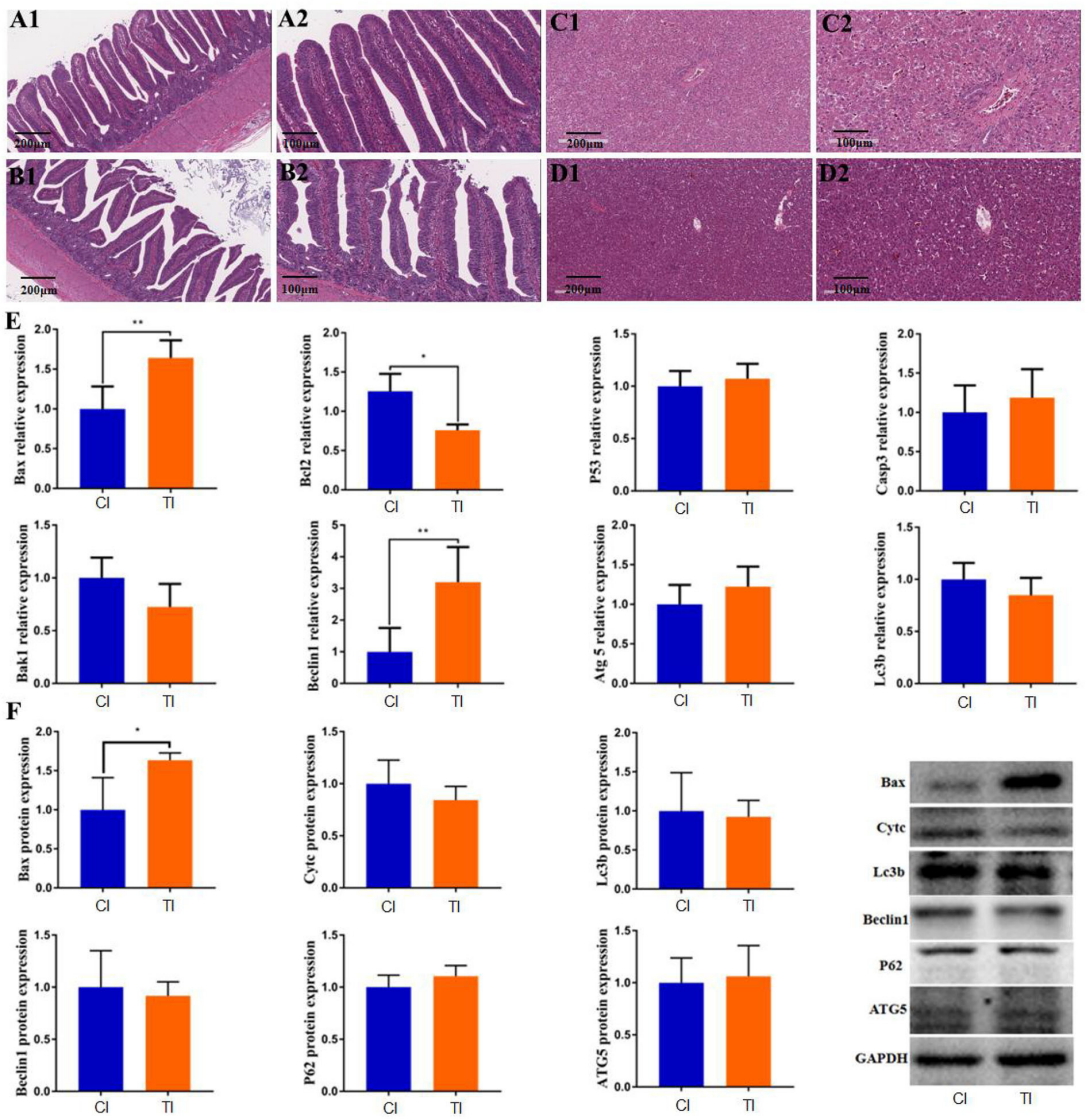
Thiram exposure caused intestinal and liver injury. **(A1,A2,B1,B2)** Histopathological observation in intestinal tissues of the control group and thiram-treated group. **(C1,C2,D1,D2)** Histopathological observation in liver tissues of the control group and thiram-treated group. **(E)** Changes in gene expression related to liver apoptosis and autophagy. **(F)** Changes in protein expression related to liver apoptosis and autophagy.

The histopathological alterations in the intestine and liver are shown in Figure 1. HE staining indicated that the intestinal structures in controls were integrated with clear borders, whereas those in thiram-treated chickens were arranged loosely, irregularly, and disorderly (Figures 1A1,A2,B1,B2). Additionally, the liver tissues in the control chickens were displayed as regular and normal structures. However, thiram exposure caused a decrease in glycogen vacuoles of hepatocytes (Figures 1C1,C2,D1,D2).

### Thiram exposure alters the expression of autophagy and apoptosis genes in liver

To investigate the effect of thiram on liver autophagy and apoptosis, we detected the expression of genes related to autophagy and apoptosis in the liver by RT-qPCR analysis. Results indicated that thiram exposure significantly affected autophagy and apoptosis-related gene expression. As shown in Figure 1E, apoptosis-related gene expressions including Bax and Bcl2 were dramatically increased in the thiram-treated group as compared to the control group, whereas no significant differences in P53, Casp3, and Bak1 levels were found between both groups. Moreover, the mRNA levels of apoptosis-related genes, such as Beclin1 in the thiram-treated group, were significantly increased *via* comparing the control group, but no obvious differences in Atg5 and Lc3b levels were observed between both groups. Similarly, the results of western blot also indicated that the expression of Bax was significantly improved in the thiram-treated group in comparison with the control group (Figure 1F).

### Sequences analysis

**Figure 2:**
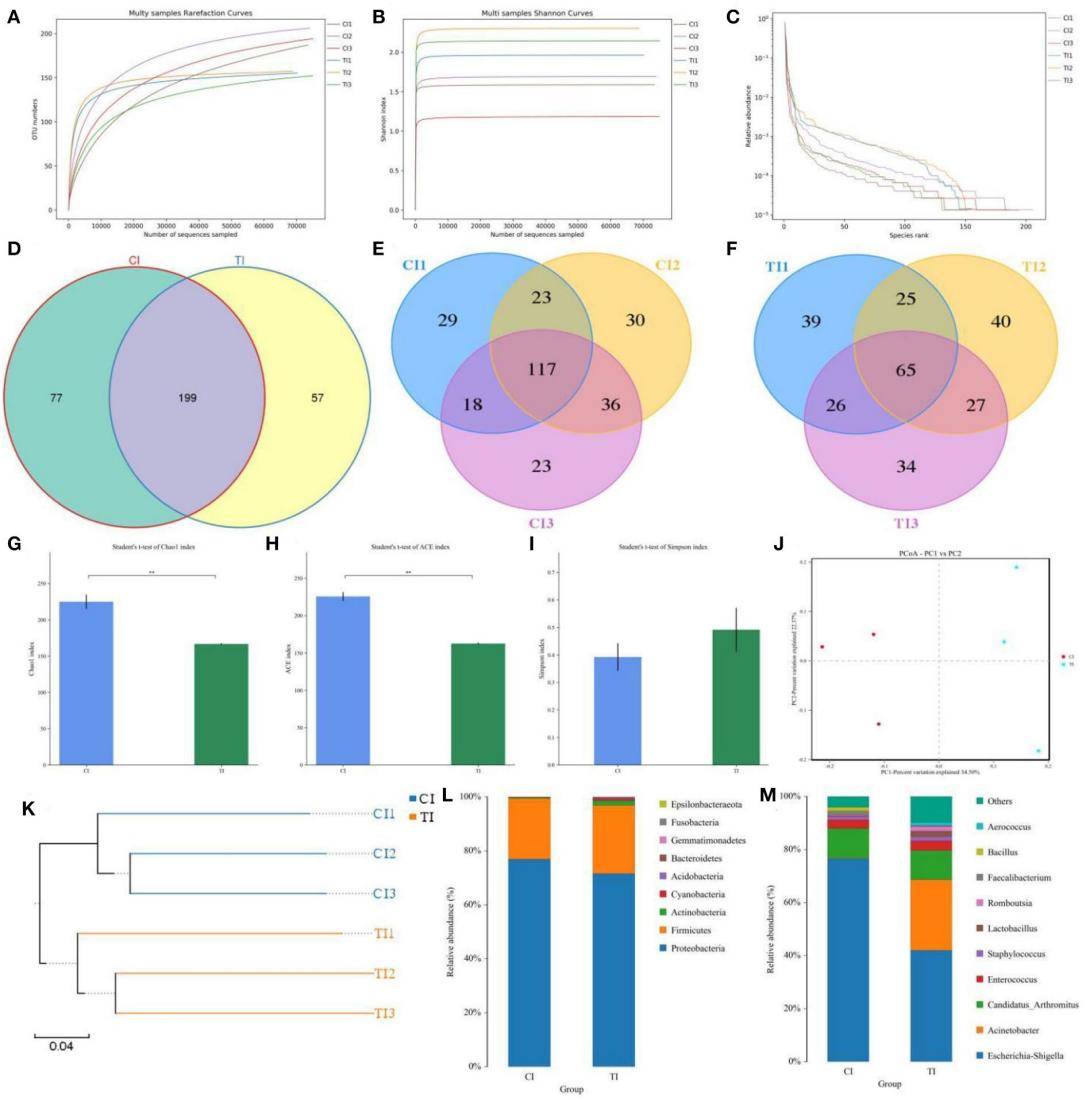
Thiram exposure changed the gut microbial composition and diversity of chickens. **(A,B)** Rarefaction curves. **(C)** Rank abundance curve. **(D–F)** Venn diagrams. **(G–I)** Chao1, ACE, and Simpson indices were employed to evaluate gut microbial alpha diversity. **(J)** PCoA map based on the unweighted UniFrac distance. **(K)** Clustering analysis. **(L,M)** Relative proportion of preponderant bacteria at the phylum and genus levels.

In this microbiome investigation, six ileal samples were conducted amplicon sequencing and 480,338 (CI = 239,765, TI = 240,573) raw sequences were collected (Table 1). After quality evaluation and data optimization, we totally acquired 458,546 (CI = 228,428, TI = 230,118) effective reads, with a median read count of 76,424 (ranging from 75,852 to 77,136) per sample. Both rarefaction and rank abundance curves of each sample supported the adequacy of the sampling efforts (Figures 2A–C). In addition, a total quantity of 333 OTUs was recognized and 199 OTUs were shared by both groups, which together made up 59.76% of the overall OTUs (Figure 2D). Moreover, 77 and 57 OTUs were uniquely recognized in CI and TI, respectively (Figures 2E,F).

**Table 1:** Sequence data of each sample.

**Sample**	**Raw reads**	**Clean reads**	**Effective reads**	**AvgLen (bp)**	**GC (%)**	**Effective (%)**
CI1	79,946	79,403	75,965	424	53.81	95.02
CI2	80,056	79,454	76,075	427	54.59	95.03
CI3	79,763	79,206	76,388	426	54.34	95.77
TI1	80,328	79,836	77,130	427	52.26	96.02
TI2	80,180	79,572	75,852	426	54.81	94.60
TI3	80,065	79,587	77,136	421	53.11	96.34

### Thiram exposure changes the gut microbial diversity

To further dissect the gut microbial alternations during thiram exposure, we calculated the alpha and beta diversity in the microbial community. There were statistically significant differences in the Chao1 (224.95 ± 9.63 vs. 166.64 ± 1.14, *p* = 0.0039) and ACE (225.6421 ± 5.8365 vs. 162.4799 ± 1.0765, *p* = 0.0004) indices, whereas the Simpson index (0.392 ± 0.05 vs. 0.4916 ± 0.0791, *p* = 0.3469) was not dramatically different between the CI and TI (Figures 2G–I). Statistical analysis of alpha diversity showed that thiram exposure significantly decreased the gut microbial richness of chickens but had no effect on the microbial diversity. Moreover, the beta diversity reflecting the differences between intergroup and intra-group individuals was evaluated using PCoA and UPGMA tree. PCoA plots revealed aggregation of intra-group samples, but a separation of samples in different groups, which was consistent with the UPGMA tree, implying that the gut microbial principal component was strongly affected by the thiram exposure (Figures 2J,K).

### Thiram exposure alters the gut microbial composition

**Figure 3:**
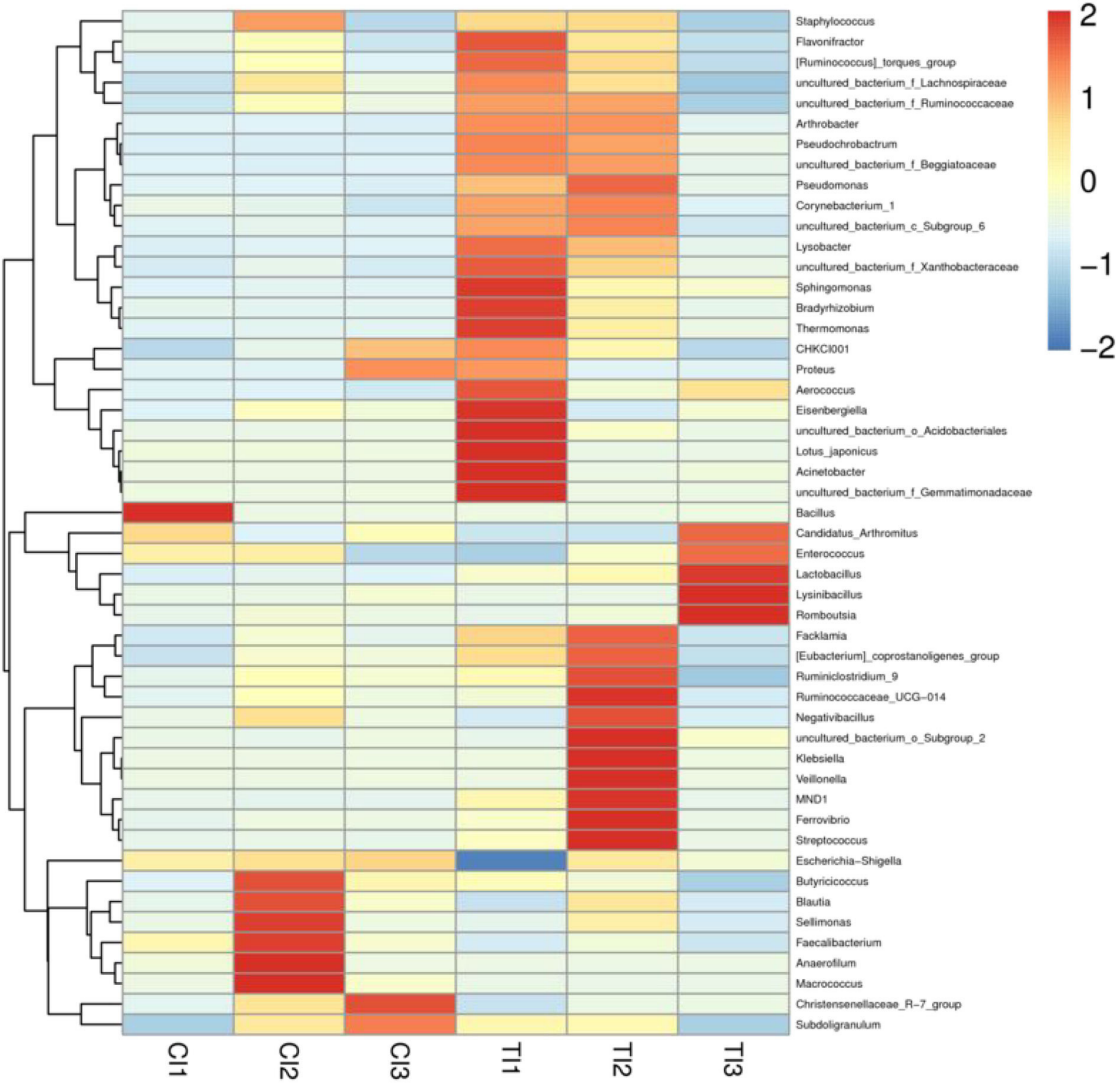
Heatmap revealing the relative abundances and distribution of the 50 most preponderant bacterial genera in both groups. The changes in blue to red of the color-block indicate the relative abundance from low to high.

The relative proportions of preponderant taxa at different levels were assessed by microbial taxon assignment, and significant variations in the gut microbial community of CI and TI were observed. There were nine phyla identified in six samples, varying from seven to nine phyla per sample. The phyla *Proteobacteria* (76.94, 71.60%), *Firmicutes* (22.52, 25.30%), and *Actinobacteria* (0.37, 1.74%) were the three most preponderant phyla in the CI and TI regardless of health status, which accounted for over 98% of all bacterial taxa (Figure 2L). Other bacterial phyla including *Gemmatimonadetes* (0.0018, 0.088%), *Fusobacteria* (0.0063, 0.069%), and *Epsilonbacteraeota* (0.00090, 0.021%) in CI and TI were indicated with lower abundances. To further dissect the influence of thiram exposure on taxonomic compositions, 168 genera were totally identified in the bacterial populations. Among them, *Escherichia–Shigella* (76.57%) was the most prevalent genus in the CI, followed by *Candidatus Arthromitus* (11.25%) and *Enterococcus* (3.15%) (Figure 2M). Moreover, *Escherichia–Shigella* (41.99%), *Acinetobacter* (26.63%), and *Candidatus Arthromitus* (11.07%) were abundantly present in the TI, which together made up approximately 80% of the bacterial composition. The heatmap reflecting the genus-level cluster analysis displayed the bacterial distribution in different samples and revealed the alternations in bacterial compositions during thiram exposure (Figure 3).

**Figure 4:**
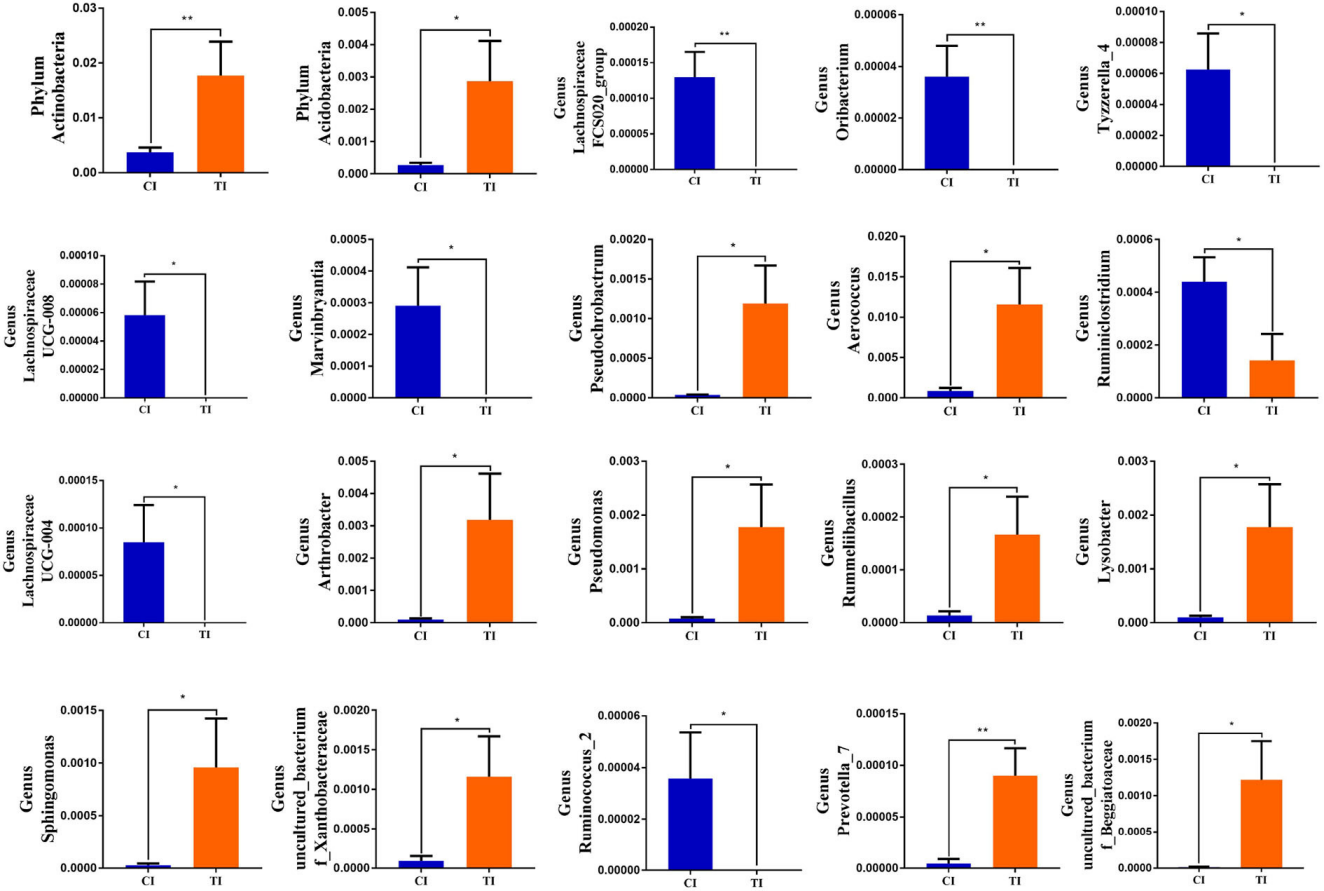
Significant shifts in the gut microbial compositions during thiram exposure. Metastats analysis displayed microbial changes between both groups. All data were represented as mean ± SD. *p < 0.05, **p < 0.01.

**Figure 5:**
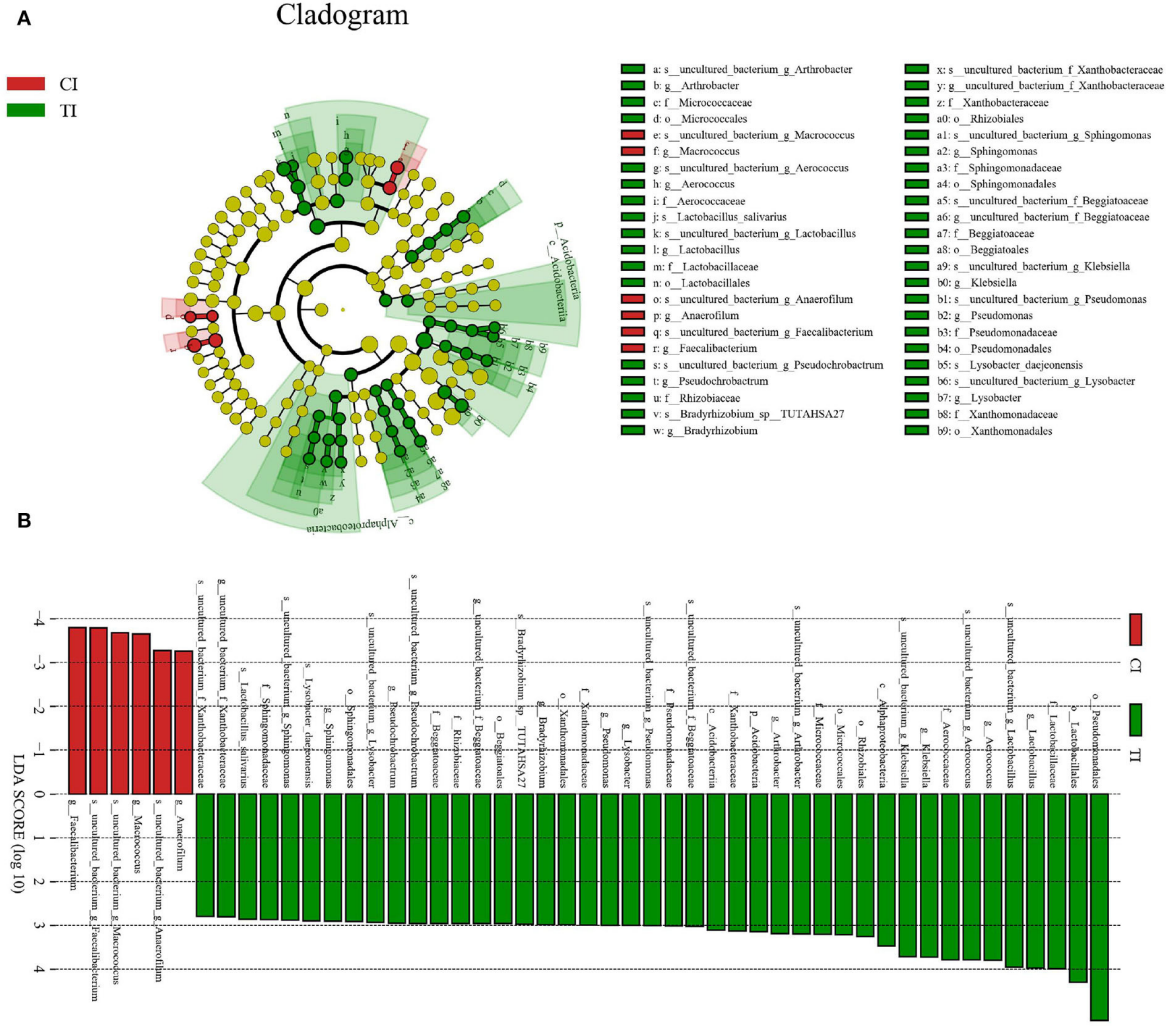
Cladogram showing the phylogenetic distribution of gut microbial community related to the control and thiram-exposure groups **(A)**. LDA scores > 2 were considered statistically significant **(B)**.

To further determine the shifts in microbial compositions of chickens during thiram exposure, Metastats analysis was conducted to characterize the differences in the gut microbiota of both groups. At the phylum level, the abundances of *Actinobacteria* (*P* < 0.01) and *Acidobacteria* (*P* < 0.05) in the TI group were dramatically dominant than in the CI group (Figure 4). At the genus level, 18 genera were totally detected to be significantly different between CI and TI groups. Among them, the relative richness of eight genera (*Lachnospiraceae_FCS020_group, Oribacterium, Tyzzerella_4, Lachnospiraceae_UCG-008, Marvinbryantia, Ruminiclostridium, Lachnospiraceae_UCG-004*, and *Ruminococcus_2*) dramatically reduced, whereas the relative abundances of 10 genera (*Prevotella_7, Pseudochrobactrum, Aerococcus, uncultured bacterium_f_Beggiatoaceae, Arthrobacter, Pseudomonas, Rummeliibacillus, Lysobacter, Sphingomonas*, and *uncultured_bacterium_f_Xanthobacteraceae*) significantly increased during thiram exposure. Among decreased bacterial genera, seven genera (*Lachnospiraceae_FCS020_group, Oribacterium, Tyzzerella_4, Lachnospiraceae_UCG-008, Marvinbryantia, Lachnospiraceae_UCG-004*, and *Ruminococcus_2*) even cannot be found in the gut microbiota of thiram-induced chicken. Given this discriminant analysis could not distinguish the dominant taxon, LEfSe was employed for generating a cladogram to identify the specific bacteria related to thiram exposure (Figure 5). We also found that *Anaerofilum, Macrococcus*, and *Faecalibacterium* were the most preponderant bacteria in the CI group, whereas *Lactobacillus, Klebsiella*, and *Bradyrhizobium* were observably overrepresented in the TI group.

## Discussion

Pesticides have been extensively used in agricultural production, but they may be accumulated and enriched in multiple ecosystems, posing a great threat to food safety and public health (Fu et al., 2018; Xu et al., 2020). Early investigations revealed that thiram exposure could cause decreased growth performance, liver toxicity, oxidative damage, and even osteogenesis disorders in chickens, but its potential influence on gut microbiota, apoptosis, and autophagy in chickens remains uncertain (Mehmood et al., 2019; Zhang et al., 2019). Here, the thiram poisoning model was constructed to investigate its influence on gut microbiota, apoptosis, and autophagy, and significant alterations of gut microbiota were observed.

Given feces cannot fully display the gut microbial abundance and diversity, we collected intestinal content for 16S rDNA amplicon sequencing. Our results indicated an observably reduced alpha diversity in the gut microbial community of chickens exposed to thiram, indicating its gut microbial dysbiosis. Typically, the gut microbial community changes dynamically within limits under the influence of age, diet, and environment and these physiological fluctuations cannot affect normal intestinal functions (Wang et al., 2018b; Li et al., 2021). However, the ecological balance of the gut microbial community may be broken and changed significantly, when the external environment shifts dramatically, including long-term exposure to antibiotics, heavy metals, and pesticides (Li et al., 2019; Zhong et al., 2021). Early investigations demonstrated that the higher gut microbial diversity and abundance were beneficial to the intestine to perform complex physiological functions and energy utilization, whereas the decreased microbial diversity may threaten the host's health (Wang et al., 2018b, 2021). Several previous studies revealed that the declined gut microbial diversity can significantly affect the metabolism of fat and carbohydrates, thereby further accelerating fat accumulation and inducing obesity and diabetes (DiBaise et al., 2008; Cani et al., 2012). Furthermore, the reduced gut microbial diversity has also been demonstrated to be closely related to the occurrence of cardiovascular diseases, diarrhea, allergies, and asthma (Tang and Hazen, 2014; Han et al., 2017). The intestine is closely associated with host immunity, metabolism, and nutrient absorption, which in turn depends on the stabilized gut microbial community (Tremaroli and Backhed, 2012; Rooks and Garrett, 2016). Therefore, imbalanced gut microbiota can also affect the immunological function and intestinal permeability of the host, which may increase morbidity (Liu et al., 2019). Moreover, gut microbial dysbiosis can impair intestinal functions and selectively promote the growth of pathogens, which may induce the occurrence of many diseases in neighbor or local organs, such as diarrhea, hepatic injury, and inflammatory bowel diseases (Frazier et al., 2011; Sheehan and Shanahan, 2017). Notably, some opportunistic pathogens that do not initially exhibit pathogenicity may also induce the occurrence of diseases, in the case of hypoimmunity and gut microbial dysbiosis (Wang et al., 2019). During gut microbial alternations, some toxic metabolites produced from pathogens can enter the intestinal hepatic circulation *via* the intestinal barrier, thereby further exacerbating the hepatic injury (Hussain et al., 2020; Zhong et al., 2021). Currently, thiram has been demonstrated to induce hepatic injury, but the potential relationship between gut microbial dysbiosis and thiram-induced liver damage remained to be investigated (Zhang et al., 2018). The results of PCoA analysis revealed that the experimental group and control group were separated from each other, suggesting an obvious difference in the gut microbial principal component between CI and TI groups. Consequently, we suspected that thiram exposure may the important driving force for shifts in the principal components of gut microbiota.

Importantly, we also observed considerable variability in some bacteria during the induction of thiram and those altered bacteria may play vital roles in the intestinal ecosystem and functions. Interestingly, some of the quantitatively reduced bacteria were considered probiotics in the intestine and seven genera even cannot be observed, suggesting that these bacteria cannot adapt to the present intestinal environment. We suspected that long-term thiram exposure disrupts the intestinal structure and environment, which inhibited the colonization of those bacteria. *Lachnospiraceae* was negatively related to intestinal inflammation (Zhao et al., 2017). *Ruminiclostridium* could improve growth performance and reduce gastrointestinal diseases (Tan et al., 2014). *Ruminococcus* is involved in the degradation of cellulose and starch (Zhao et al., 2018). Notably, the above-mentioned bacteria such as *Ruminiclostridium, Ruminococcus*, and *Lachnospiraceae* can also produce short-chain fatty acids (SCFAs). Early investigations revealed that SCFAs can inhibit the invasion and colonization of pathogenic and conditional pathogens by affecting the pH of the intestine (Van Immerseel et al., 2004; Zhou et al., 2014). Moreover, short-chain fatty acids can improve the intestinal environment (Goverse et al., 2017; Melbye et al., 2019). Recent studies on short-chain fatty acids have also shown their vital roles in alleviating inflammation, preventing cancer, regulating cell apoptosis, and lowering cholesterol (Chaudhary et al., 2021; Jiao et al., 2021). Notably, thiram exposure also resulted in a significant increase in pathogenic bacteria, such as *Aerococcus*. *Aerococcus* has been demonstrated to cause urinary tract infection and endocarditis (Yabes et al., 2018).

## Conclusion

In summary, the current study explored the alterations of gut microbiota in thiram-exposed chickens. Results indicated that thiram exposure not only obviously changed gut microbial composition and diversity but also induced liver apoptosis and autophagy. The altered gut microbiota may play crucial roles in the potential mechanism of thiram-induced intestinal toxicity and hepatotoxicity. Moreover, this research also extended the understanding of the toxicity of thiram and provided a theoretical basis for the toxicity study on prolonged thiram exposure in chickens. However, this study has some limitations including relatively small sample size and the inability to control for potentially important variables, such as individual variation and individual dietary habits.

## Data availability statement

The datasets presented in this study can be found in online repositories. The names of the repository/repositories and accession number(s) can be found below: PRJNA753238.

## Ethics statement

The animal study was reviewed and approved by Animal Welfare and Ethics Committee of South China Agricultural University.

## Author contributions

ZW and RS conceived and designed the experiments, contributed sample collection and reagents preparation, analyzed the data, and revised and reviewed the manuscript. ZW wrote the manuscript. All authors contributed to the article and approved the submitted version.

## Conflict of interest

The authors declare that the research was conducted in the absence of any commercial or financial relationships that could be construed as a potential conflict of interest.

## Publisher's note

All claims expressed in this article are solely those of the authors and do not necessarily represent those of their affiliated organizations, or those of the publisher, the editors and the reviewers. Any product that may be evaluated in this article, or claim that may be made by its manufacturer, is not guaranteed or endorsed by the publisher.
