# Supplementary material for: Pesticide thiram exposure alters the gut microbial diversity of chickens
Source: Front Microbiol. 2022 Sep 8;13:966224. doi: 10.3389/fmicb.2022.966224 (PMC9493260; doi:10.3389/fmicb.2022.966224)
Supplement: Supplementary file 1 [file Table_1.docx]

**Supplementary Table S1.** Primers used for the RT-qPCR.

| Genes name | Forward sequence (5 →3) | Reverse sequence (5 →3) |
| --- | --- | --- |
| Caspase-3 | CGGACTGTCATCTCGTTCA | TGGCTTAGCAACACACAAAC |
| Bax | TCCTCATCGCCATGCTCAT | CCTTGGTCTGGAAGCAGAAGA |
| Bcl2 | GATCGTCGCCTTCTTCGAGT | GGCCTCATACTGTTGCCGTA |
| P53 | GAGATGCTGAAGGAGATCAATGAG | GTGGTCAGTCCGAGCCTTTT |
| Beclin1 | CGTATGGCAACCACTCGTATT | TTATTGTCCCAGAAGAACCTCAG |
| ATG5 | AGAGATGTGTGGTTTGGACGC | GCCGAGGAAGGGCTGTATT |
| LC3B | AGTGAAGTGTAGCAGGATGA | AAGCCTTGTGAACGAGAT |
| Bak1 | ATGGATGCCTGTCTGTCCTGTTC | GCAGAGCAGTCCAAAGACACTGA |
| GAPDH | GCCATCACAGCCACACAGAAGA | CGGCAGGTCAGGTCAACAACAG |
